# Suppurative thyroiditis, a sign of branchiogenic fistula? Lesson based on a case report

**DOI:** 10.1016/j.radcr.2024.03.076

**Published:** 2024-05-07

**Authors:** Renato Farina, Pietro Valerio Foti, Corrado Inì, Emanuela Tona, Concetta Timpanaro, Sebastiano Galioto, Claudia Motta, Lorenzo Aliotta, Francesco Marino, Antonio Basile

**Affiliations:** Department of Medical and Surgical Sciences and Advanced Technologies “GF Ingrassia”. University of Catania, Via Santa Sofia 78. 95125 Catania, Italy

**Keywords:** Congenital anomalies, Fourth branchial cleft fistula, Thyroiditis, Ultrasound

## Abstract

Branchiogenic fistulas are congenital alterations that affect the cervical compartments. Those of the fourth branchial cleft are rarest and can begin late with very serious complications. The suppurative thyroiditis can be a complication of these alterations. We describe a case of 3-year-old girl with high fever, left cervical swelling and increased inflammation indices. The neck ultrasound showed an abscess of the left thyroid lobe and a fluid mass with aerial content in laterocervical region. On MRI, the fluid mass extended from the left piriform sinus to the mediastinum. Fluoroscopy also highlighted a fistolose trait that extended from the left side wall of the esophagus, anteriorly towards the trachea. Treatment of these pathologies must be early and a late diagnosis can put patients' lives at risk.

## Introduction

Branchiogenic cysts are congenital malformations affecting cervical compartments [1], caused by abnormal development of the branchial clefts and can clinically manifest in children or later in adults. They can be unilateral or bilateral [2], symptomatic or asymptomatic. The branchial apparatus appears between the fourth and fifth weeks of embryonic growth and consists of 4 pairs of mesodermal-origin arches developing symmetrically on each side and delimited by branchial grooves composed of ectodermal tissue. The various components of the branchial apparatus give rise to neck structures. Developmental alterations lead to branchial malformations that can manifest as cysts, sinuses, and fistulas. In detail:

a) Cysts are malformations without external communication.
b) Sinuses have an opening to the lumen of an organ or to the outside.
c) Fistulas typically connect a hollow organ to the skin tissue [3].

The branchial cleft most frequently affected by abnormalities is the second (75%) [4], followed in percentage by the first. The third and fourth are very rarely affected. Branchial cleft malformations can be classified based on their origin and location into:

A. Anomalies of the first branchial cleft, which arise within an area delimited by the chin, half of the hyoid bone, and the external auditory canal.
B. Anomalies of the second branchial cleft, which usually lie anterior to the sternocleidomastoid muscle, at the level of the hyoid bone.
C. Anomalies of the third branchial cleft, originating from the base of the piriform sinus and passing over the superior laryngeal nerve.
D. Anomalies of the fourth branchial cleft, originating from the apex of the piriform sinus, traversing the thyroid membrane, also affecting the mediastinum, and with an incidence of less than 1%. Cases of neoplastic degeneration are also reported in the literature [5]. Diagnosis is challenging due to the rarity and nonspecificity of symptoms and it is generally based on Ultrasound (US), magnetic resonance imaging (MRI), and computed tomography (CT). Patient management is very complex and must consider age and complications. The treatment of choice is surgical, involving removal of fistulous and inflammatory tissues and, if necessary, adjacent thyroid parenchyma to the piriform sinus. We describe a case of suppurative thyroiditis caused by a fourth branchial cleft fistula complicated by mediastinal abscess.

## Case presentation

**Fig. 1:**
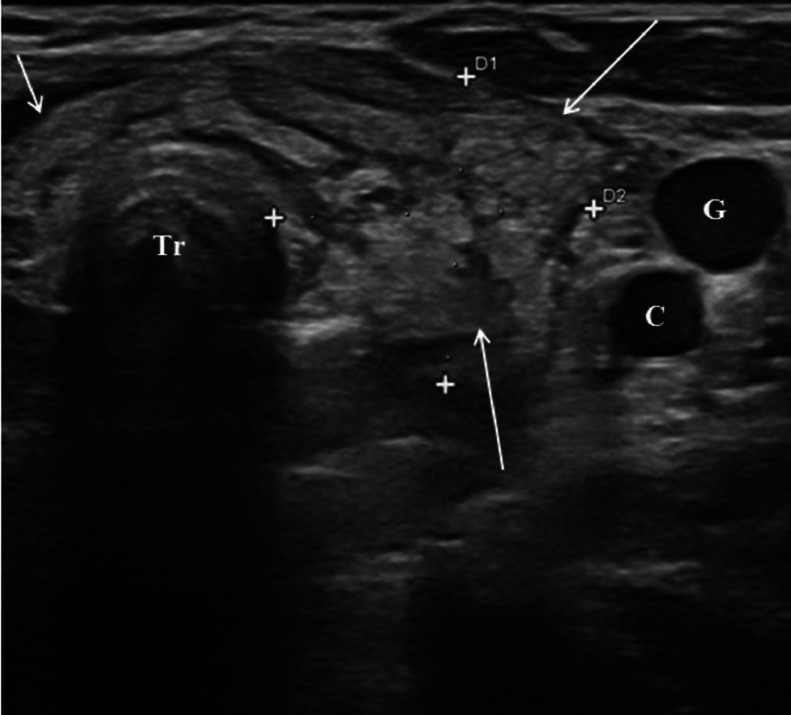
US of the neck. The cervical scans shows an increase of the left thyroid lobe (long arrows) which appears diffusely inhomogeneous. Right thyroid lobe (short arrow). Trachea (Tr). Carotid artery(C). Jugular vein (G).

**Fig. 2:**
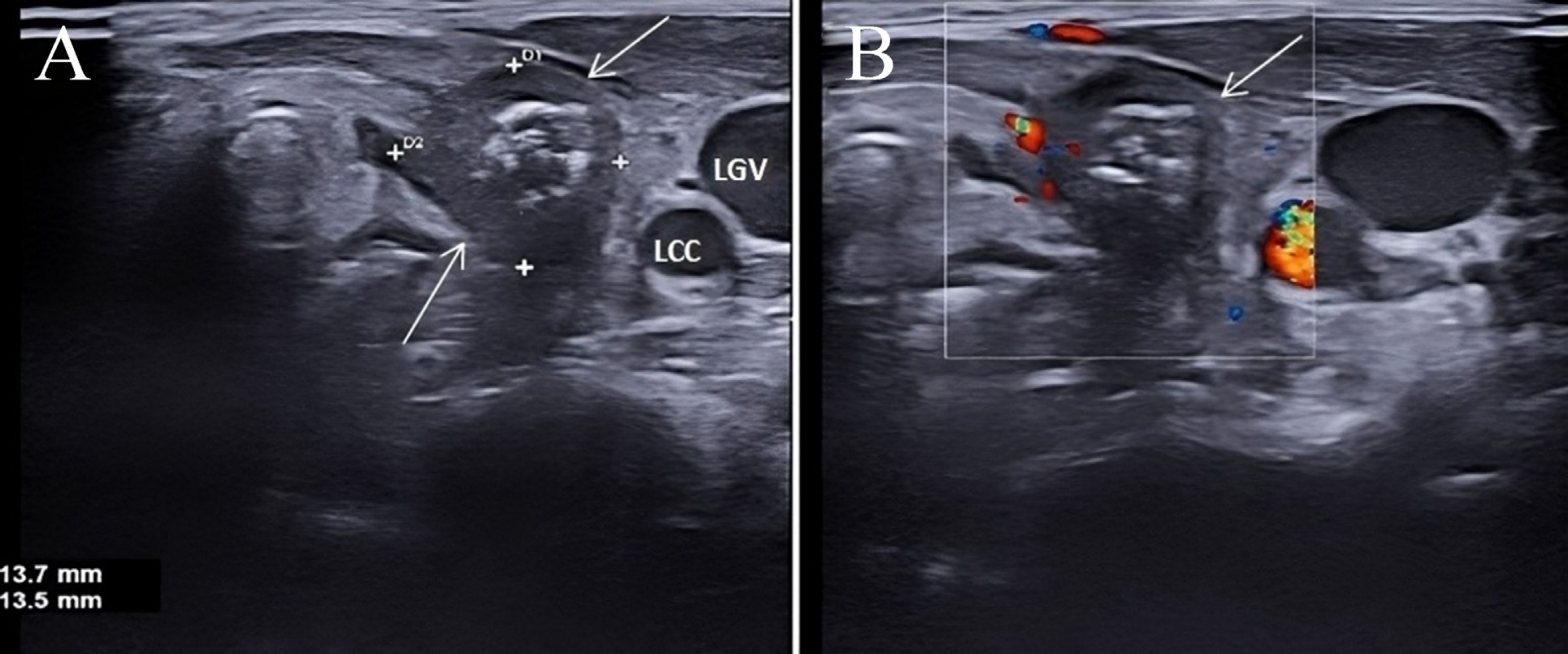
Us of the neck. Adjacent to the left thyroid lobe, gray-scale scans show (A) a rounded fluid mass with aerial content (arrows) that with (B) the color Doppler US does not shows intra -lesional vascular signals but only peripheral vascular signals that make suspects the ascessual nature of the lesion.

**Fig. 3:**
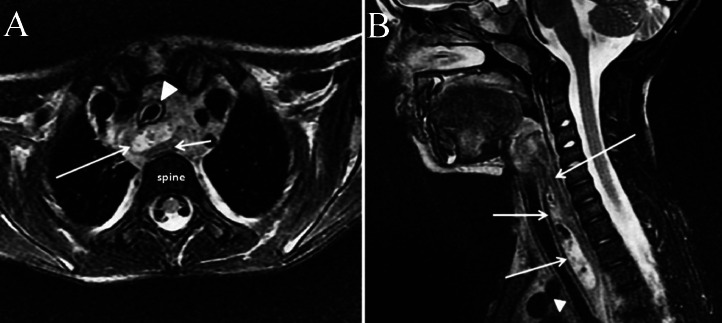
MRI of the neck. (A) In the axial view images shows an abscess (long arrow) which compresses esophagus posteriorly (short arrow) and trachea anteriorly (head arrow). (B) The sagittal view images show better the craniocaudal extent of abscess (short arrows) and the relationships it has with to the esophagus (long arrow) and trachea (arrow head).

**Fig. 4:**
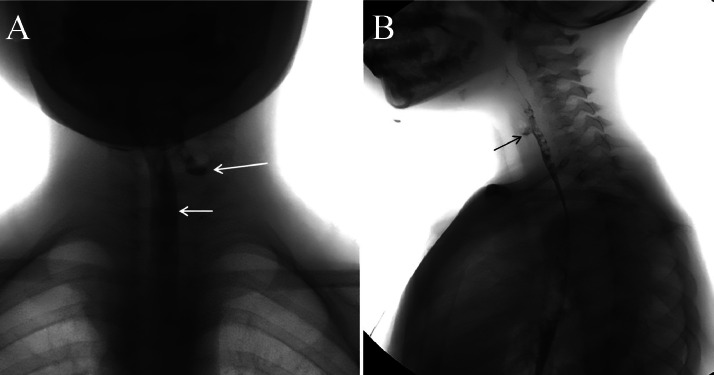
Fluoroscopy. (A) In AP view it is highlighted a contrast leak (fistula) (long arrow) originating from the left pyriform sinus which heads in the left parapharying region. Esophagus (short arrow). (B) in LL view the fistula (arrow) seems to head anteriorly towards the trachea.

A 3-year-old female patient presented to our Emergency Department with fever (39°C for 4 days), left laterocervical swelling, red and tender skin on palpation, intact sensorium and regular body weight index. Laboratory tests revealed leukocytosis 24.40 × 10^3^/µL (normal range: 5.20-12.49), increased monocytes 13.1 % (normal range: 3.4-9.0) and increased erythrocyte sedimentation rate 28 mm/h (normal range: 0-10). Ultrasound (US), magnetic resonance imaging (MRI), and fluoroscopy were performed. US examination was conducted using the May Lab Nine device (Esaote Biomedica, Genoa) with an 8-15 MHz linear probe. MRI was performed using a 1.5 T unit device (Signa HDxT; GE Healthcare, Milwaukee, WI, USA). US revealed an enlarged and heterogeneous left thyroid lobe (Fig. 1), compared to the contralateral lobe, along with a rounded, fluid-filled mass in the left laterocervical region, containing intralesional air and absence of vascular signals (Figs. 2A and B). The US study was performed by an operator with 20 years of experience in the field. MRI revealed a fluid-filled mass with air component which extended from the left piriform sinus to the left laterocervical region and proceeding into the mediastinum between the trachea and esophagus up to the sixth thoracic vertebra (Figs. 3A and B). Subsequently, fluoroscopy revealed contrast medium leakage from the left piriform sinus into the parapharyngeal region (Figs. 4A and B). The patient underwent incision and drainage of the abscess under open neck surgery, endoscopic cauterization, and antibiotic treatment with penicillin, leading to symptom regression after 2 weeks.

## Discussion

During embryonic development, branchial cleft gives rise to various structures including the laryngeal cartilages, the ultimobranchial body from which originate the follicular cells secreting thyroid hormone and the superior parathyroid glands, the superior laryngeal nerve, the left thoracic aorta, the constrictor muscles of the larynx and pharynx, and the proximal right subclavian artery.

Anatomically, the fistulas of the fourth branchial cleft originate from the apex of the pyriform sinus, traveling inferiorly along the tracheoesophageal sulcus, behind the thyroid gland up to the thorax.

Malformations of the fourth branchial cleft are rare and can clinically manifest, in advanced cases, with mediastinal abscesses and/or acute suppurative thyroiditis. Depending on their size, branchial cysts and fistulas can compress adjacent vascular structures, salivary glands, and the esophagus. The left side is generally more affected, although the reason has not been yet clarified; according to some hypotheses, it could be due to a more complex course of the fourth branchial cleft or the absence or involution of the right fourth branchial cleft. Differential diagnosis should be made with third branchial cleft fistulas, neck tumors, metastases, lymphadenopathy, ectopic thyroid, and salivary glands. Imaging is essential to demonstrate all alterations, and the choice of the most appropriate methods is crucial for the correct diagnosis and staging of the disease. US is the first-level examination and can detect thyroid inflammation and abscesses in the cervical region. In the case we report, US was very useful for recognizing thyroid and neck abscesses. Since suppurative thyroiditis is generally a late manifestation of fourth branchial cleft fistula, we also extended the study to the mediastinum using MRI, which allowed for a correct diagnosis. After locating the fistula origin with MRI, it is equally important to document its course for subsequent surgical treatment; for this purpose fluoroscopy can easily identify the fistula course, avoiding the need for CT scans which would result in a higher exposure of ionizing radiation. Patient management is complex and must consider their age and complications. The optimal therapeutic approach always involves surgical removal of all branchial cleft malformations [6] except for acute suppurative thyroiditis and/or mediastinal abscesses, for which surgery should be preceded by antibiotic therapy, surgical incision, and/or abscess drainage. These latter treatments usually do not resolve the issue and are associated with high recurrence rates; therefore, they should always be followed by surgical excision of the fistula. In our case, abscess drainage and antibiotic therapy led to symptom resolution within 2 weeks, and we decided to postpone surgery as it is advisable to perform the procedure after the age of 4 when the facial nerve will be sufficiently developed, reducing the risk of paralysis. Fourth branchial cleft malformations [7] are rare and difficult to diagnose due to the nonspecificity of symptoms and should be suspected in cases of suppurative thyroiditis associated with cervical abscess. The combination of US [8], MRI and fluoroscopy represents the best compromise for identifying all abnormalities. Describing these rare pathologies can significantly contribute to reducing false negatives. Failure to diagnose can expose patients to serious health risks.

## Ethical approval

``All procedures performed in studies involving human participants were in accordance with the ethical standards of the institutional and/or national research committee and with the 1964 Helsinki declaration and its later amendments or comparable ethical standards.

## Author Contribution

RF Study design/planning collected data, preparation of manuscript, data analysis/statistics, data interpretation and involved in project development, literature analysis/search PVF, CI, ET, CT, SG, CM, LA, and FM collected data, wrote the manuscript, literature analysis/search. RF and AB: wrote the manuscript.

## Patient consent

The consent was obtained from the patient for the publication of this case report and accompanying images.
